# The Complete Chloroplast Genome Sequence of the Medicinal Plant *Swertia mussotii* Using the PacBio RS II Platform

**DOI:** 10.3390/molecules21081029

**Published:** 2016-08-09

**Authors:** Beibei Xiang, Xiaoxue Li, Jun Qian, Lizhi Wang, Lin Ma, Xiaoxuan Tian, Yong Wang

**Affiliations:** 1 School of Chinese Materia Medica, Tianjin University of Traditional Chinese Medicine, Anshan Road 312, Tianjin 300193, China; lzhwang_2009@163.com (L.W.); malin7983@163.com (L.M.); 2 College of Life Science, Nankai University, Weijin Road 94, Tianjin 300071, China; m15822139395@163.com; 3 Institute of Medicinal Plant Development, Chinese Academy of Medical Sciences and Peking Union Medical College, Malianwa North Road 151, Beijing 100193, China; qianjun@ymail.com; 4 Tianjin State Key Laboratory of Modern Chinese Medicine, Tianjin University of Traditional Chinese Medicine, Anshan Road 312, Tianjin 300193, China

**Keywords:** *Swertia mussotii*, medicinal plant, chloroplast genome, PacBio RS, Gentianaceae

## Abstract

*Swertia mussotii* is an important medicinal plant that has great economic and medicinal value and is found on the Qinghai Tibetan Plateau. The complete chloroplast (cp) genome of *S. mussotii* is 153,431 bp in size, with a pair of inverted repeat (IR) regions of 25,761 bp each that separate an large single-copy (LSC) region of 83,567 bp and an a small single-copy (SSC) region of 18,342 bp. The *S. mussotii* cp genome encodes 84 protein-coding genes, 37 transfer RNA (tRNA) genes, and eight ribosomal RNA (rRNA) genes. The identity, number, and GC content of *S. mussotii* cp genes were similar to those in the genomes of other Gentianales species. Via analysis of the repeat structure, 11 forward repeats, eight palindromic repeats, and one reverse repeat were detected in the *S. mussotii* cp genome. There are 45 SSRs in the *S. mussotii* cp genome, the majority of which are mononucleotides found in all other Gentianales species. An entire cp genome comparison study of *S. mussotii* and two other species in Gentianaceae was conducted. The complete cp genome sequence provides intragenic information for the cp genetic engineering of this medicinal plant.

## 1. Introduction

*Swertia mussotii* Franch (Zang Yin Chen, in Tibetan medicine) belongs to the family Gentianaceae. This species grows on the Qinghai Tibetan Plateau at an elevation of 3800–5000 m. To date, several pharmaceutically-active compounds have been isolated and structurally identified from the whole *S. mussotii* plant, including oleanolic acid, ursolic acid, mangiferin, swertiamarin, and gentiopicroside [1,2,3,4]. Modern pharmacological research has demonstrated that these compounds have anti-hepatitis activity [5,6,7]. Due to the overexploitation of this plant, *S. mussotii* as a wild resource has become rare. *S. mussotii* seeds only germinate poorly when planted at low elevations.

Chloroplasts originated from the interaction of photosynthetic bacteria with non-photosynthetic hosts through endosymbiosis [8]. Chloroplasts are photosynthetic organelles that synthesise starch, amino acids, pigments, and fatty acids [9,10]. The chloroplast has its own genome, and a typical circular cp genome is composed of four parts: a large single-copy (LSC) region, a small single-copy (SSC) region, and two inverted repeat (IR) regions. The majority of angiosperm cp genomes are highly conserved in gene content and order [11]. However, large-scale genome rearrangement and gene loss have been identified in several angiosperm lineages [12,13].

The third-generation sequencing platform, PacBio, based on single-molecule, real-time (SMRT) sequencing technology, generates average read lengths of over 10 kb, with half of the reads over 20 kb and a maximum read length reaching up to 60 kb, using the newest P6-C4 chemical reagents on the current PacBio RS II machine. In addition to its extraordinarily long read length, this platform provides uniform coverage across GC-abnormal regions because no PCR amplification is required during the library construction [14,15]. Many concerns have concentrated on the high rates of random error in single-pass reads (approximately 11% to 14%) [15]. However, this can be improved given sufficient sequencing depth [15]. Additionally, the optimisation of the PacBio assembly algorithm [16,17,18] has made this platform widely applied in de novo genome sequencing [19,20], as well as full-length transcriptome sequencing [21,22], for a growing number of species.

Due to the low GC content and the IR regions, it is difficult to use short reads from second-generation sequencing to recover a single contig spanning the whole cp genome [14]. Using PacBio, long reads can greatly reduce the complexity of the assembly, and PacBio has already been successfully applied in many chloroplast genome sequencing projects, including *Ananas comosus* var. *comosus* [23], *Aconitum barbatum* var. *puberulum* [24], *Beta vulgaris* [25], and *Gentiana straminea* [26]. Meanwhile, comparative studies among the three generations of sequencing technologies (Sanger, Illumina and PacBio) have demonstrated the reliability and accuracy of SMRT sequencing [27,28].

Currently, more than 1000 complete cp genome sequences have been deposited in the NCBI Organelle Genome Resources [29]. However, few reports have been published on the genetic diversity of cpDNA from Gentianaceae [26]. The chloroplast genome sequences of two members of the Gentianaceae, *Gentiana straminea* [26] and *Gentiana crassicaulis*, have been analysed. Here, we report the complete cp genome sequence of *S. mussotii* as determined using PacBio technology. Comparative sequence analysis was conducted among published Gentianaceae cp genomes.

## 2. Results and Discussion

### 2.1. Features of the S. mussotii Chloroplast Genome

**Figure 1:**
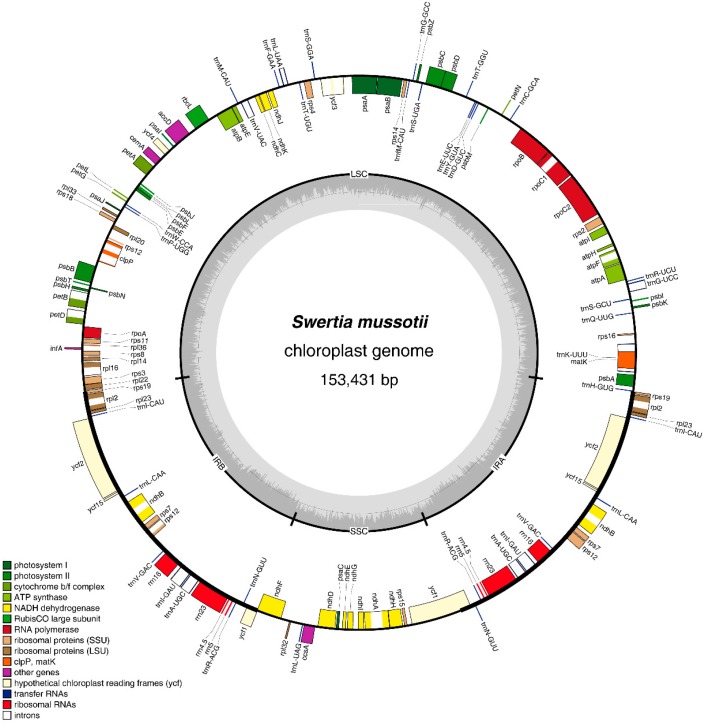
Gene map of the *S. mussotii* chloroplast genome. Genes drawn inside the circle are transcribed clockwise, and those outside are counterclockwise. Genes are colour-coded based on the functional groups to which they belong. CDS: protein-coding regions.

The complete cp genome of *S. mussotii* is 153,431 bp in size, with a pair of IR regions of 25,761 bp that separate an LSC region of 83,567 bp from an SSC region of 18,342 bp (Table 1 and Figure 1). The overall GC content of the *S. mussotii* cp genome is 38.2%, with the IR regions possessing higher GC content (43.5%) than the LSC (36.2%) and SSC regions (31.9%) (Table 1). The high GC content of the IR regions is caused by the high GC content of the four ribosomal RNA (rRNA) genes (55.2%) present in this region [30]. The *S. mussotii* cp genome encodes 84 protein-coding genes, 37 transfer RNA (tRNA) genes, and eight rRNA genes (Table 2). Seven protein-coding, seven tRNA, and all rRNA genes are duplicated in the IR regions. The non-coding regions constitute 41.6% of the genome, including introns, pseudogenes, and intergenic spacers; coding regions constitute 58.4%.

There are five pseudogenes, i.e., *accD*, *rps16*, *infA*, *rps19*, and *ycf1*. The *accD* gene in *S. mussotii* contains internal stop codons. The *accD* gene also exists as a pseudogene in *Jasminum nudiflorum* and *Trachelium caeruleum*, but it is a normal gene in *G. straminea*. The *rps16* gene lacks exon 2, a phenomenon that has been observed in related species. In *S. mussotii*, *rps16* is a pseudogene, whereas in *Syzygium cumini*, *Eucalyptus globulus*, and *Gossypium barbadense*, the *rps16* gene encodes a 16S ribosomal protein [31]. The absence or incompleteness of this gene has also been reported in other plants [32,33]. The *infA* gene is 3′ truncated, though it is a normal gene in many other cp genomes [34,35].

The *S. mussotii* cp genome has 17 intron-containing genes, of which three (*clpP*, *rps12*, and *ycf3*) contain two introns (Table 3). The *rps12* gene is a trans-spliced gene with the 5′ end located in the LSC region and the duplicated 3′ end located in the IR regions. *trnK-UUU* has the largest intron, which contains the *matK* gene. Together, all of the genes of *S. mussotii* are encoded by 25,731 codons. Among these, leucine, with 2769 (10.7%) of the codons, is the most frequent amino acid in the genome, and cysteine, with 292 (1.1%), is the least frequent (Table 4). Within the protein-coding regions (CDS), the percentages of AT content for the first, second, and third codon positions are 54.3%, 61.3%, and 68.8%, respectively. The bias towards a higher AT representation at the third codon position has also been observed in other plant cp genomes [36,37].

### 2.2. Repeat Analysis

**Figure 2:**
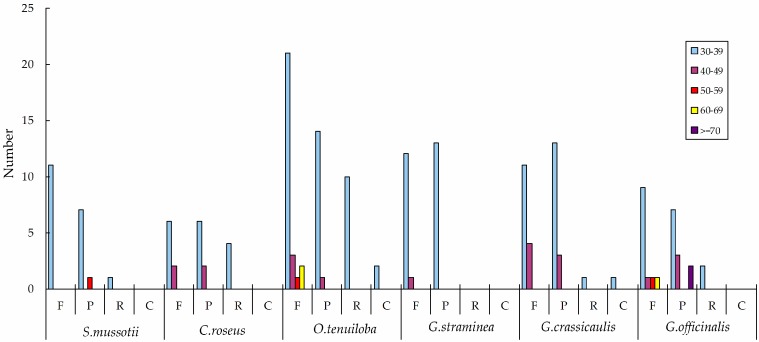
Repeat sequences in six Gentianales chloroplast genomes. REPuter was used to identify repeat sequences with length ≥ 30 bp and sequence identify ≥90% in the chloroplast genomes. F, P, R, and C indicate the repeat types F (forward), P (palindrome), R (reverse), and C (complement), respectively. Repeats with different lengths are indicated in different colours.

Repeat structure analysis revealed the presence of 11 forward repeats, eight palindromic repeats, and one reverse repeat in the *S. mussotii* cp genome (Table 5). The repeats were mostly distributed in the intergenic spacer (IGS) and intron sequences. We analysed the repeats of several other species in Gentianales (Figure 2). Interestingly, this comparison revealed that the longest repeats in the five Gentianales cp genomes were 30–39 bp, and the *Oncinotis tenuiloba* cp genome contained the greatest total number of repeats (54). Chloroplast simple sequence repeats (SSRs) have been accepted as effective molecular markers [38,39]. There were 45 SSRs in the *S. mussotii* cp genome (Table 6), the majority of which were mononucleotides (30) that we found in all the other species [40]. Pentanucleotides and hexanucleotides were rarely found in the Gentianales cp genomes (Table 7). Most SSR loci were located in LSC regions. In all species, the majority of the tri- to hexanucleotides were AT-rich. An average of 62% of all SSRs in the Gentianales cp genomes were A/T mononucleotides. These results are consistent with the view that SSRs in cp genomes contribute to AT richness [41,42].

### 2.3. Comparative Chloroplast Genomic Analysis

The whole cp genome sequence of *S. mussotii* was compared to those of *G. straminea* and *G. crassicaulis*. The cp genome of *S. mussotii* is the longest of the three cp genomes, measuring approximately 4.4 kb and 4.7 kb longer than those of *G. straminea* and *G. crassicaulis*, respectively. There are no significant differences in sequence length between the SSCs or the IRs, and the variation in sequence length is mainly attributable to the difference in the length of the LSC region (Table S2) [40].

**Figure 3:**
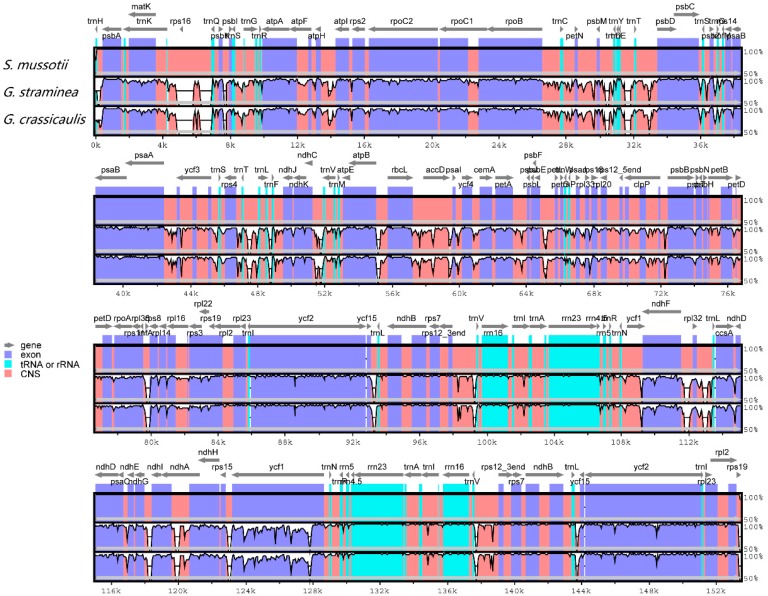
Comparison of three chloroplast genomes using mVISTA. Grey arrows and thick lines above the alignment indicate genes with their orientation and the position of the IRs, respectively. A cut-off of 70% identity was used for the plots, and the y-axis represents the percent identity between 50%–100%. Genome regions are color-coded as protein-coding (exon), rRNA, tRNA, and conserved noncoding sequences (CNS).

The overall sequence identity of the three Gentianaceae cp genomes was plotted using mVISTA, with the annotation of *S. mussotii* as a reference (Figure 3). The comparison shows that the two IR regions are less divergent than the LSC and SSC regions. Additionally, the coding regions are more conserved than the non-coding regions [26], and the highly divergent regions among the three cp genomes occur in the non-coding regions, including *ndhD-ccsA*, *ndhI-ndhG*, and *trnH-psbA*. Similar results have been observed in other plant cp genomes [26,43]. In our study, we observed that all four rRNA genes are the most conserved, while the most divergent coding regions are the *clpP*, *rpl22*, *ycf1*, *rpl32*, *ycf15*, and *matK* genes. The divergent portions of non-coding regions of cp genomes have proven useful for phylogenetic analysis [44,45].

### 2.4. IR Contraction and Expansion

**Figure 4:**
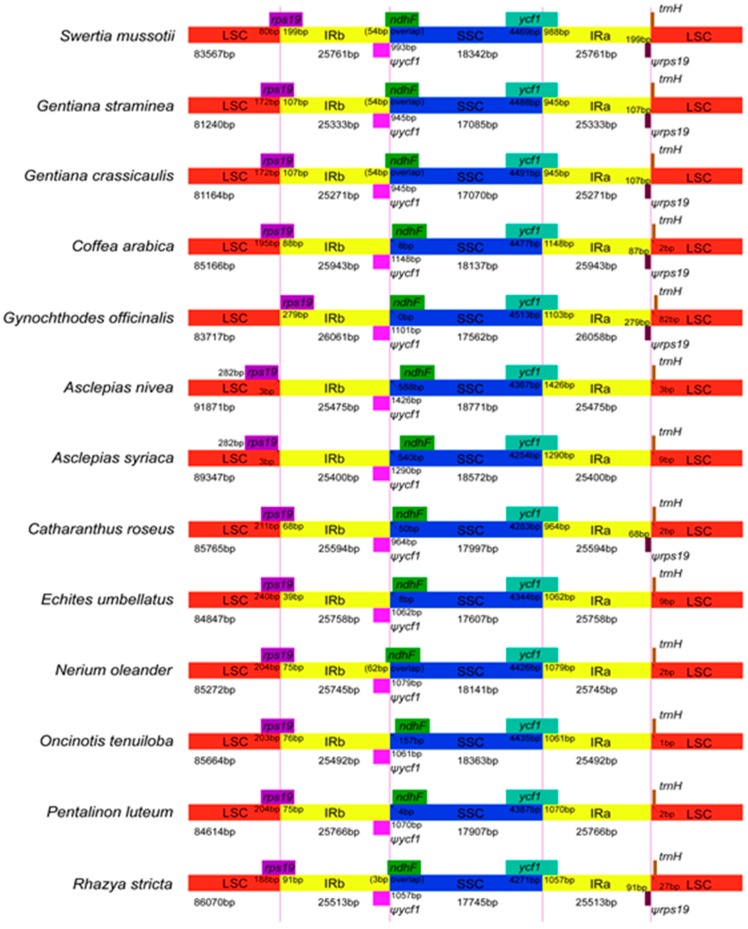
Comparison of the borders of the LSC, SSC, and IR regions among thirteen chloroplast genomes. Ψ indicates a pseudogene. This figure is not to scale.

IR contraction was observed at the junction of the IR and LSC regions of the *S. mussotii* cp genome. This contraction has also been found in the twelve species of Gentianales analysed (*G. straminea*, *G. crassicaulis*, *C. arabica*, *C. roseus*, *A. nivea*, *A. syriaca*, *R. stricta*, *E. umbellatus*, *N. oleander*, *O. tenuiloba*, *P. luteum*, and *G. officinalis*) (Figure 4). In all of these species, the IRA/SSC junction is situated in the coding region of the *ycf1* gene, resulting in the duplication of the 3′ end of this gene. This duplication produces a pseudogene of variable length at the IRB/SSC border. The lengths of the *ycf1* pseudogenes varied from 945 bp to 1426 bp. In addition, the *ycf1* pseudogene and the *ndhF* gene overlapped in *S. mussotii*, *G. straminea*, *G. crassicaulis*, *N. oleander*, and *R. stricta* by 54 bp, 54 bp, 54 bp, 62 bp, and 3 bp, respectively. The IRb/LSC border is located in the coding region of *rps19* in all the compared plants, except for *A. nivea*, *A. syriaca*, and *G. officinalis*. *rps19* pseudogenes of various lengths were also found at the IRa/LSC borders in *S. mussotii*, *G. straminea*, *G. crassicaulis*, *C. arabica*, *C. roseus*, *G. officinalis*, and *R. stricta*. *S. mussotii* had the longest *rps19* pseudogene, at 199 bp in length. The *trnH* genes of these thirteen species were all located in the LSC region, 0–82 bp away from the IRa/LSC border. In the cp genome, the IR/LSC boundaries are not static, but are subject to a dynamic and random processes that allow conservative expansions and contractions [46].

## 3. Materials and Methods

### 3.1. DNA Sequencing, Genome Assembly, and Validation

Fresh leaves were collected from *S. mussotii* in Yushu County, Qinghai Province. Total DNA was extracted using the NuClean PlantGen DNA Kit (CWBIO, Beijing, China) and was used to construct an SMRT sequencing library with an insert size of 10 kb. The genome was sequenced using the PacBio RS II platform (Pacific Biosciences, Menlo Park, CA, USA) at the Institute of Medicinal Plant Development of the Chinese Academy of Medical Sciences. We assembled the cp genome of *S. mussotii* as follows: first, the PacBio reads were error-corrected and assembled to produce the initial contigs using the hierarchical genome assembly process (HGAP) of SMRT Analysis (Pacific Biosciences); then, the coverage for each contig was calculated by mapping the PacBio reads to these initial contigs using BLASR [47], and contigs either showing similarity to the closely-related cp genome sequences or exhibiting similar coverage were extracted; finally, the complete cp genome was constructed by assembling these contigs. Based on the BLASR results, 3904 PacBio reads were used in the assembly of the complete cp genome, with a total length of 46,037,271 bp, thus yielding a 300× depth of the cp genome. Four junction regions between IRs and LSC/SSC were verified by PCR amplifications and Sanger sequencing. The final cp genome of *S. mussotii* was submitted to GenBank under the accession number KU641021.

### 3.2. Genome Annotation and Codon Usage

DOGMA [48] was used to annotate the cp genome, followed by manual corrections. The tRNA genes were identified using tRNAscan-SE [49]. The circular genome map was drawn using OGDRAW [50]. Codon usage and GC content were analysed using MEGA5 [51].

### 3.3. Genome Comparison and Repeat Analyses

mVISTA [52,53] was used to compare the cp genome of *S. mussotii* with two other cp genomes using the annotation of *S. mussotii* as a reference.

Repeats (forward, palindromic, reverse, and complement) and simple sequence repeats (SSRs) were identified using REPuter [54] and MISA, respectively, with the same parameters as described in Ni et al. [26].

## 4. Conclusions

This is the first study to analyse the complete cpDNA sequence of *S. mussotii*. The chloroplast genome structure and composition of *S. mussotii* are similar to those reported for other Gentianaceae. In addition, the distributions and locations of repeated sequences were determined. All of these repeats, together with the aforementioned SSRs, are informative sources for the exploration of new molecular markers. Studying the cp genome facilitates the identification of the optimal intergenic spacers for transgene integration and the development of site-specific cp transformation vectors in chloroplast genetic engineering. To date, many transgenes have been successfully introduced into the plastid genomes of the tobacco model species and of selected other important crop plants [55,56]. The feasibility of metabolic engineering in transgenic plastids has been demonstrated for several nutritionally important biochemical pathways, including carotenoid biosynthesis [57] and fatty acid biosynthesis [58,59]. With the details of the bioactive compound synthesis pathway in *S. mussotii* having been described [60], there is no doubt that plastid engineering holds great potential in secondary metabolic engineering to enhance the production of pharmaceutically active compounds.

## Figures and Tables

**Table 1:** Base composition in the *S. mussotii* chloroplast genome.

Region		T (U) (%)	C (%)	A (%)	G (%)	Length (bp)
LSC		32.6	18.5	31.2	17.7	83,567
SSC		34.1	16.3	34.0	15.6	18,342
IRa		28.3	22.5	28.2	21.0	25,761
IRb		28.2	21.0	28.3	22.5	25,761
Total		31.3	19.3	30.5	18.8	153,431
CDS		31.3	18.1	30.2	20.4	77,193
	1st position	23.9	19.2	30.4	26.5	25731
	2nd position	32.6	20.6	28.7	18.1	25731
	3rd position	37.2	14.6	31.6	16.6	25731

**Table 2:** Genes present in the *S. mussotii* chloroplast genome.

No.	Group of Genes	Gene Names
1	Photosystem I	*psaA*, *psaB*, *psaC*, *psaI*, *psaJ*
2	Photosystem II	*psbA*, *psbB*, *psbC*, *psbD*, *psbE*, *psbF*, *psbH*, *psbI*, *psbJ*, *psbK*, *psbL*, *psbM*, *psbN*, *psbT*, *psbZ*
3	Cytochrome b/f complex	*petA*, *petB* *, *petD* *, *petG*, *petL*, *petN*
4	ATP synthase	*atpA*, *atpB*, *atpE*, *atpF* *, *atpH*, *atpI*
5	NADH dehydrogenase	*ndhA* *, *ndhB* * (×2), *ndhC*, *ndhD*, *ndhE*, *ndhF*, *ndhG*, *ndhH*, *ndhI*, *ndhJ*, *ndhK*
6	RuBisCO large subunit	*rbcL*
7	RNA polymerase	*rpoA*, *rpoB*, *rpoC1* *, *rpoC2*
8	Ribosomal proteins (SSU)	*rps2*, *rps3*, *rps4*, *rps7* (×2), *rps8*, *rps11*, *rps12* ** (×2), *rps14*, *rps15*, *rps18*, *rps19*
9	Ribosomal proteins (LSU)	*rpl2* * (×2), *rpl14*, *rpl16* *, *rpl20*, *rpl22*, *rpl23* (×2), *rpl32*, *rpl33*, *rpl36*
10	Other genes	*clpP* *, *matK*, *ccsA*, *cemA*
11	Proteins of unknown function	*ycf1*, *ycf2* (×2), *ycf3* **, *ycf4*, *ycf15* (×2)
12	Transfer RNAs	37 tRNAs (6 contain one intron each, 7 in the IRs)
13	Ribosomal RNAs	*rrn4.5* (×2), *rrn5* (×2), *rrn16* (×2), *rrn23* (×2)

The presence of one or two asterisks after the name of a gene indicates that that gene contains one or two introns, respectively.

**Table 3:** The genes with introns in the *S. mussotii* chloroplast genome and the lengths of the exons and introns.

Gene	Location	Exon I (bp)	Intron I (bp)	Exon II (bp)	Intron II (bp)	Exon III (bp)
*atpF*	LSC	161	700	403		
*clpP*	LSC	71	784	292	680	228
*ndhA*	SSC	561	1117	540		
*ndhB*	IR	777	683	756		
*petB*	LSC	6	727	642		
*petD*	LSC	8	678	475		
*rpl16*	LSC	9	764	399		
*rpl2*	IR	393	657	435		
*rpoC1*	LSC	435	734	1623		
rps12 *	LSC	114	-	232	535	26
*trnA-UGC*	IR	38	824	35		
*trnG-UCC*	LSC	23	689	48		
*trnI-GAU*	IR	37	950	35		
*trnK-UUU*	LSC	37	2496	35		
*trnL-UAA*	LSC	37	374	50		
*trnV-UAC*	LSC	38	601	37		
*ycf3*	LSC	126	745	228	770	153

* The *rps12* gene is a trans-spliced gene with the 5′ end located in the LSC region and the duplicated 3′ end in the IR region.

**Table 4:** The codon-anticodon recognition pattern and codon usage for the *S. mussotii* chloroplast genome.

Amino Acid	Codon	No.	RSCU	tRNA	Amino Acid	Codon	No.	RSCU	tRNA
Phe	UUU	981	1.32		Tyr	UAU	738	1.59	
Phe	UUC	507	0.68	*trnF-GAA*	Tyr	UAC	189	0.41	*trnY-GUA*
Leu	UUA	847	1.84	*trnL-UAA*	Stop	UAA	49	1.75	
Leu	UUG	551	1.19	*trnL-CAA*	Stop	UAG	21	0.75	
Leu	CUU	610	1.32		His	CAU	467	1.5	
Leu	CUC	187	0.41		His	CAC	157	0.5	*trnH-GUG*
Leu	CUA	392	0.85	*trnL-UAG*	Gln	CAA	698	1.54	*trnQ-UUG*
Leu	CUG	182	0.39		Gln	CAG	207	0.46	
Ile	AUU	1047	1.47		Asn	AAU	920	1.5	
Ile	AUC	435	0.61	*trnI-GAU*	Asn	AAC	303	0.5	*trnN-GUU*
Ile	AUA	660	0.92	*trnI-CAU*	Lys	AAA	988	1.45	*trnK-UUU*
Met	AUG	582	1	*trn(f)M-CAU*	Lys	AAG	377	0.55	
Val	GUU	510	1.45		Asp	GAU	802	1.61	
Val	GUC	187	0.53	*trnV-GAC*	Asp	GAC	194	0.39	*trnD-GUC*
Val	GUA	528	1.5	*trnV-UAC*	Glu	GAA	923	1.45	*trnE-UUC*
Val	GUG	185	0.52		Glu	GAG	350	0.55	
Ser	UCU	540	1.6		Cys	UGU	221	1.52	
Ser	UCC	352	1.04	*trnS-GGA*	Cys	UGC	70	0.48	*trnC-GCA*
Ser	UCA	382	1.13	*trnS-UGA*	Stop	UGA	14	0.5	
Ser	UCG	221	0.66		Trp	UGG	461	1	*trnW-CCA*
Pro	CCU	395	1.42		Arg	CGU	339	1.28	*trnR-ACG*
Pro	CCC	234	0.84		Arg	CGC	102	0.39	
Pro	CCA	318	1.14	*trnP-UGG*	Arg	CGA	356	1.35	
Pro	CCG	166	0.6		Arg	CGG	139	0.53	
Thr	ACU	485	1.46		Arg	AGA	385	1.14	*trnR-UCU*
Thr	ACC	272	0.82	*trnT-GGU*	Arg	AGG	143	0.42	
Thr	ACA	413	1.24	*trnT-UGU*	Ser	AGU	477	1.81	
Thr	ACG	157	0.47		Ser	AGC	171	0.65	*trnS-GCU*
Ala	GCU	614	1.8		Gly	GGU	534	1.2	
Ala	GCC	225	0.66		Gly	GGC	198	0.45	*trnG-GCC*
Ala	GCA	378	1.11	*trnA-UGC*	Gly	GGA	705	1.59	*trnG-UCC*
Ala	GCG	148	0.43		Gly	GGG	342	0.77	

RSCU: Relative Synonymous Codon Usage.

**Table 5:** Repeat sequences and their distribution in the *S. mussotii* chloroplast genome.

No.	Size (bp)	Type	Repeat 1 Start	Repeat 1 Location	Repeat 2 Start	Repeat 2 Location	Region
1	39	F	97971	IGS (*rps12*, *trnV-GAC*)	119586	*ndhA* (intron)	IRb, SSC
2	38	F	44377	*ycf3* (intron 1)	97971	IGS (*rps12*, *trnV-GAC*)	LSC, IRb
3	38	F	44377	*ycf3* (intron 1)	119586	*ndhA* (intron)	LSC, SSC
4	37	F	216	IGS (*trnH-GUG*, *psbA*)	244	IGS (*trnH-GUG*, *psbA*)	LSC
5	38	F	39302	*psaB* (*CDS*)	41526	*psaA* (CDS)	LSC
6	32	F	8154	*trnS-GCU*	36099	*trnS-UGA*	LSC
7	30	F	7704	IGS (*psbK*, *psbI*)	28958	IGS (*petN*, *psbM*)	LSC
8	30	F	9536	*trnG-UCC*	37013	*trnG-GCC*	LSC
9	30	F	38751	*psaB* (CDS)	40966	*psaA* (CDS)	LSC
10	30	F	58479	*ΨaccD*	58512	*ΨaccD*	LSC
11	30	F	75545	*petB* (intron)	138996	IGS (*trnV-GAC*, *rps12*)	LSC, IRa
12	51	P	114672	IGS (*ccsA*, *ndhD*)	114675	IGS (*ccsA*, *ndhD*)	SSC
13	39	P	119586	*ndhA* (intron)	138988	IGS (*trnV-GAC*, *rps12*)	SSC, IRa
14	38	P	44377	*ycf3* (intron 1)	138989	IGS (*trnV-GAC*, *rps12*)	LSC, IRa
15	32	P	8154	*trnS-GCU*	45722	*trnS-GGA*	LSC
16	32	P	36096	*trnS-UGA*	45725	*trnS-GGA*	LSC
17	30	P	44378	*ycf3* (intron 1)	75545	*petB* (intron)	LSC
18	30	P	75545	*petB* (intron)	119587	*ndhA* (intron)	LSC, SSC
19	30	P	75545	*petB* (intron)	97972	IGS (*rps12*, *trnV-GAC*)	LSC, IRb
20	31	R	42871	IGS (*psaA*, *ycf3*)	42875	IGS (*psaA*, *ycf3*)	LSC

F = forward, P = palindrome, IGS = intergenic spacer.

**Table 6:** Simple sequence repeats in the *S. mussotii* chloroplast genome.

Unit	Length	No.	SSR Start	Region
A	16	1	68265	LSC
	13	3	45315	LSC
			80949	LSC
			114240	SSC
	11	1	22183	LSC
	10	7	8410	LSC
			12227	LSC
			57572	LSC
			63341	LSC
			71135	LSC
			77632	LSC
			122496	SSC
C	11	1	60812	LSC
T	14	1	60823	LSC
	13	2	118296	SSC
			118428	SSC
	12	4	5757	LSC
			32886	LSC
			35984	LSC
			112141	SSC
	11	3	1828	LSC
			124064	SSC
			125507	SSC
	10	7	92	LSC
			7909	LSC
			54930	LSC
			66001	LSC
			120007	LSC
			125752	LSC
			127189	SSC
AT	10	1	47791	LSC
TA	10	1	47617	LSC
ATT	15	1	119656	LSC
TTA	12	1	127046	LSC
TTC	12	1	35761	LSC
TTG	12	1	111418	SSC
AATT	16	1	29843	LSC
ATTT	12	1	116917	SSC
CATA	12	1	151279	IRa
TATG	12	1	85709	IRb
TATT	12	1	116932	SSC
TGTC	12	1	30554	LSC
TAATA	15	1	116944	SSC
TATTG	15	1	62151	LSC
CCTTTA	18	1	37196	LSC

**Table 7:** Distribution of SSRs present in the Gentianales chloroplast genomes.

Taxon	Genome Size (bp)	AT (%)	SSR Type						CDS			
			Mono	Di	Tri	Tetra	Penta	Hexa	Total	% ^a^	No. ^b^	% ^c^
*Swertia mussotii*	153,431	62	30	2	4	6	2	1	45	58	10	22
*Gentiana straminea*	148,991	62	27	3	2	7	0	0	39	61	10	26
*Gentiana crassicaulis*	148,776	62	27	4	2	7	0	1	41	61	10	24
*Coffea arabica*	155,189	63	31	5	3	4	0	0	43	59	8	19
*Catharanthus roseus*	154,950	62	33	6	7	9	1	0	56	59	5	9
*Asclepias nivea*	161,592	62	47	15	6	23	3	4	98	56	17	17
*Asclepias syriaca*	158,719	62	56	13	7	16	2	7	101	55	17	17
*Rhazya stricta*	154,841	62	33	5	9	12	3	0	62	58	6	10
*Echites umbellatus*	153,970	62	47	9	7	7	1	1	72	59	7	10
*Nerium oleander*	154,903	62	42	6	3	8	2	0	61	59	10	16
*Oncinotis tenuiloba*	155,011	62	41	7	4	9	2	0	63	58	5	8
*Pentalinon luteum*	154,053	62	34	5	2	5	3	0	49	57	6	12
*Gynochthodes officinalis*	153,398	62	26	4	7	3	4	1	45	60	7	16

CDS: coding regions. ^a^ Percentages were calculated using the total length of the CDS divided by the genome size. ^b^ Total number of SSRs identified in the CDS. ^c^ Percentages were calculated using the total number of SSRs in the CDS divided by the total number of SSRs in the genome.

## Supplementary Materials

Supplementary materials can be accessed at: http://www.mdpi.com/1420-3049/21/8/1029/s1.
