# Supplementary material for: The Complete Chloroplast Genome Sequence of the Medicinal Plant Swertia mussotii Using the PacBio RS II Platform
Source: Molecules. 2016 Aug 9;21(8):1029. doi: 10.3390/molecules21081029 (PMC6274542; doi:10.3390/molecules21081029)
Supplement: Supplementary file 1 [file molecules-21-01029-s001.pdf]

Supplementary Materials: The Complete Chloroplast Genome Sequence of the Medicinal Plant *Swertia mussoti* Using the PacBio RS II Platform

Beibei Xiang, Xiaoxue Li, Jun Qian, Lizhi Wang, Lin Ma, Xiaoxuan Tian and Yong Wang

Table S1. The list of accession numbers of the chloroplast genome sequences used in this study.

No.	Taxon	Family	Order	GenBank Accession Number
1	*Swertia mussotii*	Gentianaceae	Gentianales	KU_641021
2	*Gentiana straminea*	Gentianaceae	Gentianales	NC_027441
3	*Gentiana crassicaulis*	Gentianaceae	Gentianales	NC_027442
4	*Asclepias nivea*	Apocynaceae	Gentianales	NC_022431
5	*Asclepias syriaca*	Apocynaceae	Gentianales	NC_022432
6	*Catharanthus roseus*	Apocynaceae	Gentianales	NC_021423
7	*Echites umbellatus*	Apocynaceae	Gentianales	NC_025655
8	*Nerium oleander*	Apocynaceae	Gentianales	NC_025656
9	*Oncinotis tenuiloba*	Apocynaceae	Gentianales	NC_025657
10	*Pentalinon luteum*	Apocynaceae	Gentianales	NC_025658
11	*Rhazya stricta*	Apocynaceae	Gentianales	NC_024292
12	*Coffea arabica*	Rubiaceae	Gentianales	NC_008535
13	*Gynochthodes officinalis*	Rubiaceae	Gentianales	NC_028009
14	*Salvia miltiorrhiza*	Lamiaceae	Lamiales	NC_020431
15	*Olea europaea*	Oleaceae	Lamiales	NC_013707
16	*Jasminum nudiflorum*	Oleaceae	Lamiales	NC_008407
17	*Boea hygrometrica*	Gesneriaceae	Lamiales	NC_016468
18	*Sesamum indicum*	Pedaliaceae	Lamiales	NC_016433
19	*Nicotiana tabacum*	Solanaceae	Solanales	NC_001879
20	*Capsicum annuum*	Solanaceae	Solanales	NC_018552
21	*Datura stramonium*	Solanaceae	Solanales	NC_018117
22	*Solanum lycopersicum*	Solanaceae	Solanales	NC_007898
23	*Ipomoea batatas*	Convolvulaceae	Solanales	NC_026703
24	*Atropa belladonna*	Solanaceae	Solanales	NC_004561
25	*Ageratina adenophora*	Asteraceae	Asterales	NC_015621
26	*Campanula takesimana*	Campanulaceae	Asterales	NC_026203
27	*Chrysanthemum indicum*	Asteraceae	Asterales	NC_020320
28	*Helianthus annuus*	Asteraceae	Asterales	NC_007977
29	*Lactuca sativa*	Asteraceae	Asterales	NC_007578
30	*Jacobaea vulgaris*	Asteraceae	Asterales	NC_015543
31	*Aralia undulata*	Araliaceae	Apiales	NC_022810
32	*Brassaiopsis hainla*	Araliaceae	Apiales	NC_022811
33	*Daucus carota*	Apiaceae	Apiales	NC_008325
34	*Eleutherococcus senticosus*	Araliaceae	Apiales	NC_016430
35	*Panax ginseng*	Araliaceae	Apiales	NC_006290
36	*Cucumis sativus*	Cucurbitaceae	Cucurbitales	NC_007144
37	*Arabidopsis thaliana*	Brassicaceae	Brassicales	NC_000932

Table S2. Size comparison of *Swertia mussotii* chloroplast genomic regions with three other Gentianaceae chloroplast genomes.

Species	Length (bp)			
	Total genome	LSC	SSC	IR
Swertia mussotii	153,431	83,567	18,342	25,761
Gentiana straminea	148,991	81,240	17,085	25,333
Gentiana crassicaulis	148,776	81,164	17,070	25,271
